# Changes in transcriptional pausing modify the folding dynamics of the pH-responsive RNA element

**DOI:** 10.1093/nar/gkt868

**Published:** 2013-09-26

**Authors:** Gal Nechooshtan, Maya Elgrably-Weiss, Shoshy Altuvia

**Affiliations:** Department of Microbiology and Molecular Genetics, IMRIC, The Hebrew University-Hadassah Medical School, Jerusalem 91120, Israel

## Abstract

Previously, we described a novel pH-responsive RNA element in *Escherichia coli* that resides in the 5′ untranslated region of the *alx* gene and controls its translation in a pH-dependent manner. Under normal growth conditions, this RNA region forms a translationally inactive structure, but when transcribed under alkaline conditions, it forms an active structure producing the Alx protein. We identified two distinct transcriptional pause sites and proposed that pausing at these sites interfered with the formation of the inactive structure while facilitating folding of the active one. Alkali increases the longevity of pausing at these sites, thereby promoting folding of the translationally active form of *alx* RNA. We show here that mutations that modify the extent and/or position of pausing, although silent with regard to structure stability *per se*, greatly influence the dynamics of folding and thereby translation. Our data illustrate the mechanistic design of *alx* regulation, relying on precise temporal and spatial characteristics. We propose that this unique design provides an opportunity for environmental signals such as pH to introduce structural changes in the RNA and thereby modulate expression.

## INTRODUCTION

RNA conformation plays a major role in processes such as transcription termination, RNA processing and translation initiation. Hence, the folding of RNA is an important determinant in regulation of gene expression in many cellular systems, in both prokaryotes and eukaryotes. Furthermore, the structure of RNAs may change in response to environmental signals. Thus, understanding the mechanisms by which RNAs assemble to form active units is of considerable interest.

An increasing body of evidence suggests that much of the regulation of gene expression that is based on RNA folding is accomplished during the elongation phase of transcription and involves pausing of RNA polymerase (RNAP) (1). Pausing, a temporal inhibition of the transcription elongation complex, plays an essential role in coupling transcription and translation in bacteria (2). Pausing also allows interaction with, or recruitment of regulators (3,4) and ligands and acts as the first step leading to transcription termination (5–8). Transcriptional pausing has also been shown to assist in the correct folding of certain *Escherichia coli* non-coding RNAs, including signal recognition particle (SRP), RNase P and transfer-messenger RNA (tmRNA) (9). Importantly, pausing plays a crucial role in gene expression regulation by riboswitches, coordinating RNA folding with ligand binding (10–13).

**Figure 1.**
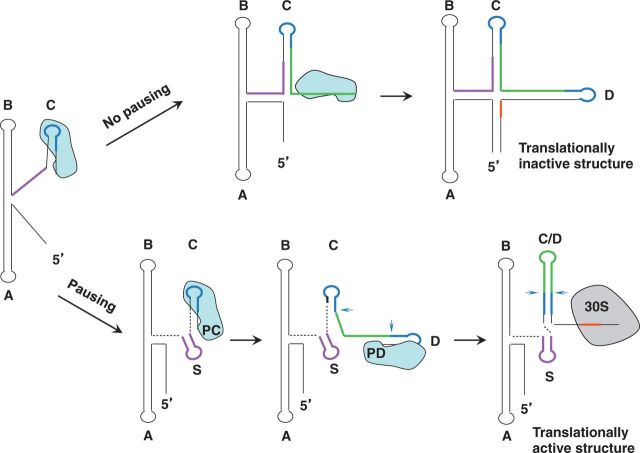
A model for PRE*-alx* regulation by transcriptional pausing [adapted from (14)]. In the absence of transcriptional pausing, PRE*-alx* forms a translationally inactive structure, whereas when folding is affected by RNAP pausing, the newly synthesized RNA adopts an active structure in which the ribosome-binding site (in red) is exposed for 30 S ribosomal subunit binding. Formation of the active structure involves pausing of RNAP at two sites located at hairpin C (PC) and hairpin D (PD). On pausing of RNAP (shaded in light blue) at PC, the sequence required to complete stem C is sequestered within the transcription elongation complex and thus formation of hairpin C is prevented. Instead, hairpin S (indicated by a purple line) forms. Similarly, pausing at PD inhibits the formation of hairpin D, allowing base pairing between the complementary sequences in hairpins C and D (in blue) and formation of the apical part of hairpin C/D (in green).

Recently, we have described the first example of a pH-responsive RNA regulator that operates in the absence of a ligand, undergoing a conformational change depending on RNAP pausing. This pH-responsive RNA element (PRE) precedes the *alx* gene, encoding a putative transporter, and controls its translation in a pH-dependent manner. In cells grown under neutral conditions, the PRE-*alx* transcript forms a translationally inactive structure, whereas growth under high pH results in the formation of an active structure and production of Alx (14). The pH-dependent folding of PRE-*alx* RNA into an active structure requires *de novo* synthesis, during which RNAP, by pausing at two sites, plays an active role in controlling which structure forms. The location of the pause sites led us to suggest that both correlate with the formation of the *alx* translatable structure. By pausing at the first site (PC in Figure 1), RNAP sequesters part of the sequence that is required to form hairpin C and thereby allows formation of an alternative hairpin (hairpin S in Figure 1). Once formed, this hairpin precludes annealing of the former one. Similarly, by pausing at the second site (PD), the elongation complex prevents the formation of hairpin D and thereby indirectly promotes annealing of complementary sequences in hairpins C and D, forming a new hairpin (hairpin C/D). As a consequence, the alternative translationally active structure forms. In this study, we show that mutations that change the precise location of pausing or mutations leading to increased pausing have a profound effect on the folding path of PRE-*alx*.

## MATERIALS AND METHODS

### Bacterial growth conditions

*Escherichia coli* MC4100 cells were grown at 37°C (200 rpm) in Luria-Bertani broth (LB) medium (pH 6.8) or in potassium modified LB medium (LBK; pH 8.4) (14). Ampicillin (100 µg/ml) and kanamycin (40 µg/ml) were added where appropriate.

### Plasmid construction

The construction of plasmids carrying P*_alx_*-PRE-*alx*'-'*lacZ* (pSA60; translation fusion), P_T7_-PRE*-alx'* in pGEM4 (pSA66), P*_alx_*-PRE-*alx*' in pGEM4 (pSA64) and P*_alx_*-PRE-*alx*' in pZE12 (pSA65) has been described previously (14,15).

### Site-directed mutagenesis

Mutants A_132_G and C_135_U were generated by PCR using plasmid carrying P*_alx_*-PRE-*alx*' (pSA64) as template and two tail-to-tail divergent primers (5′ phosphorylated) of which one carried the desired mutation. The PCR product was subjected to blunt-end ligation and then used as template to amplify the desired fragment for sub-cloning. To construct P*_alx_*-PRE-*alx*' deleted for bases 12–91 of PRE (pSA64 Δ12–91), divergent primers spaced by the site of the deletion were used instead of tail to tail. Mutant A_137_G was constructed in two steps using overlapping divergent primers, both carrying the desired mutation. The first two PCR reactions contained each, one of the primers, the corresponding fragment end primer used to generate the wild-type fragment and plasmid carrying P*_alx_*-PRE*-alx'-'lacZ* (pSA60) as template. The products from these two reactions were used as template in a second PCR reaction containing the fragment end primers used to generate the wild-type fragment. The resulting fragment was then sub-cloned.

### β-galactosidase assays

Overnight cultures were diluted 1:100 in fresh LB or LBK medium and grown to OD_600_ of 0.4. β-galactosidase activity was assayed as described (16).

### *In vitro* structure probing of RNA generated by T7 RNAP

RNA was synthesized from pSA66 (wild-type and mutants) and probed as described previously (14).

### Native gels

RNA synthesized from pSA66 (wild-type and mutants) was incubated for 30 min at 22°C and then analyzed on 5% non-denaturing polyacrylamide gels as described previously (14).

### Cotranscriptional *in vitro* structure probing

RNA was synthesized using *E. coli* RNAP (1 U; Epicentre) at pH 7.2 in 50 µl reactions containing 20 mM Tris–HCl, 150 mM KCl, 10 mM MgCl_2_, 5 mM dithiothreitol (DTT), NTPs (250 µM each), 20 units RNase inhibitor (Takara Bio) and 2.5 µg of plasmid DNA carrying P*_alx_*-PRE*-alx'* (pSA64; wild-type and mutants). The mixture was preincubated for 5 min at 37°C before the addition of enzyme. After 5 min of synthesis at 37°C, 1.7 µl of dimethyl sulfate (DMS) (diluted 1:10 in ethanol) was added. The reactions were stopped 5 min after the addition of DMS by phenol/chloroform extraction followed by ethanol precipitation in the presence of 0.3 M sodium acetate and 1 µl of Quick-Precip (Edge BioSystems). To detect the modified sites, the treated RNA was annealed with 0.6 pmol end-labeled primer (70°C for 10 min, followed by incubation for 5–10 min on ice) and then subjected to primer extension (at 43°C for 60 min) in 15 µl reactions containing 0.5% (v/v) Tween 20, 50 mM Tris–HCl (pH 8.3), 40 mM KCl, 5 mM MgCl_2_, 10 mM DTT, deoxynucleotide triphosphate (dNTPs) (1 mM each) and 25 units Expand reverse transcriptase (Roche). The extension products were separated on 6% polyacrylamide, 7.8 M urea gels, alongside sequencing reactions.

### Single round transcription

Single round transcription reactions were performed at pH 7.2 as described in (17) with slight modifications. To allow formation of halted elongation complexes and incorporation of label, 1.25 µg of plasmid DNA carrying P*_alx_*-PRE*-alx'* (pSA64; wild-type and mutants) was incubated at 37°C for 10 min in the presence of 10 µCi of α-^32 ^P-ATP, 20 mM Tris–HCl, 150 mM KCl, 10 mM MgCl_2_, 2.77 mM DTT, 1 µM ATP, 2.5 µM GTP, 20 µg/ml BSA, 150 µM GpC dinucleotide (Sigma), glycerol to 3.33% (v/v) (including glycerol of the enzyme storage buffer) and 0.5 unit *E. coli* RNAP in a 45-µl volume. Thereafter, aliquots of 9 µl were transferred to pre-warmed tubes, and transcription was resumed by adding 1 µl of pre-warmed solution containing NTPs (2.5 mM each), 20 mM Tris–HCl, 150 mM KCl, 10 mM MgCl_2_ and 150 µg/ml rifampicin (to block re-initiation). The reactions were stopped by freezing in liquid nitrogen. Single round transcription reactions with PCR generated templates were performed similarly, with the following modifications—reactions were conducted at 30°C (Supplementary Figure S2) or 25°C (Figure 4b), the incubation time for formation of halted elongation complexes was 15 min (Supplementary Figure S2) or 20 min (Figure 4b) and 225 ng of template and 1 unit of enzyme were used. Samples were analyzed on 6% polyacrylamide and 7.8 M urea gels. The position of PC in reactions using PCR generated templates was verified by comparison with reactions using plasmid templates (Supplementary Figure S2). Band intensities were measured using the Quantity One program (Bio-Rad).

### *In vivo* DMS structure probing

The structure of PRE-*alx*' RNA (wild-type and mutants) was probed *in vivo* in LB medium as described previously for pSA65 (14). Detection of modified sites was performed with 50 µg of total RNA for each sample.

## RESULTS

### Mutations designed to modify pausing at PRE affect *alx* translation

**Figure 2.**
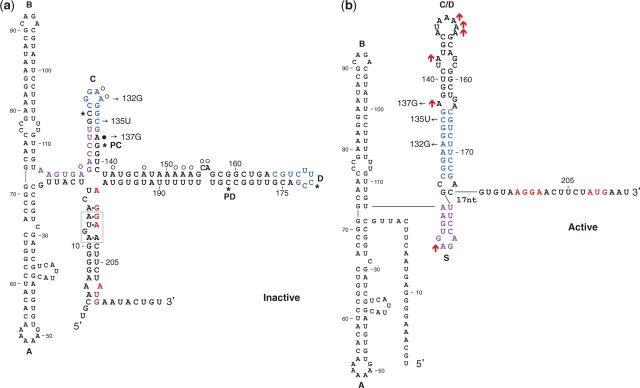
Translationally inactive (**a**) and active (**b**) structures. The Shine–Dalgarno and the initiation codon of *alx* are in red. Complementary sequences in hairpins C and D are in blue. Thin arrows indicate specific nucleotide changes generated by site-directed mutagenesis. To form the translationally active structure, the proximal part of hairpin C folds into the small hairpin S (nucleotides marked in purple), whereas basepairing between loops C and D forms stem C/D. A loop E motif in the inactive structure is boxed (dashed line). PC and PD denote the pause sites at hairpins C and D, respectively. Asterisks in (a) delineate the regions typically sequestered within the paused transcription elongation complex. The cotranscriptional structure probing data obtained on synthesis with *E. coli* RNAP (Figure 5) is presented on the structures. (a) Data obtained with wild-type RNA. Circles indicate strong (filled circles) and weak (open circles) DMS modification sites. (b) Data obtained with the high-level expression mutant A_132_G. Red upward arrows indicate increased modification intensity compared with wild-type. The two structures, the inactive and the active one, were previously shown to form under neutral and high pH conditions, respectively.

The nature of the sequence located upstream of a pause site greatly influences RNAP transcriptional elongation (18–20). To learn about the relationship between pausing and RNA folding, we introduced single point mutations (A_132_G, C_135_U and A_137_G) upstream of the first pause site, which were expected to affect pausing efficiency and/or position. The mutations designed were assumed to have a minimal effect, or none at all, on the stability of either the active or the inactive structure (Figure 2). The mutations A_132_G, C_135_U and A_137_G were first analyzed for their effect on expression using P*_alx_*-PRE*-alx'-'lacZ* translation fusions. These assays showed that the mutations substantially increased the reporter expression level compared with wild-type. The response of these mutants to high pH was more moderate than that of wild-type (Table 1).

**Table 1.** Mutations modifying pause C affect *alx* expression

Translation fusion^a^	β-galactosidase units		Position^b^
	pH 6.8	pH 8.4	
Wild-type	68 ± 17	531 ± 182	138
A_132_G	182 ± 27	1243 ± 163	138
C_135_U	424 ± 5	1708 ± 225	141
A_137_G	178 ± 30	885 ± 111	140

^a^Translation fusion plasmids carry the *alx* promoter followed by PRE and the first 53 nucleotides of *alx* (P*_alx_*-PRE*-alx'-'lacZ*). Results (in Miller units) are displayed as mean ± standard deviation.

^b^The numbers indicate the estimated location of pause C.

### Structural analysis of the mutants

**Figure 3.**
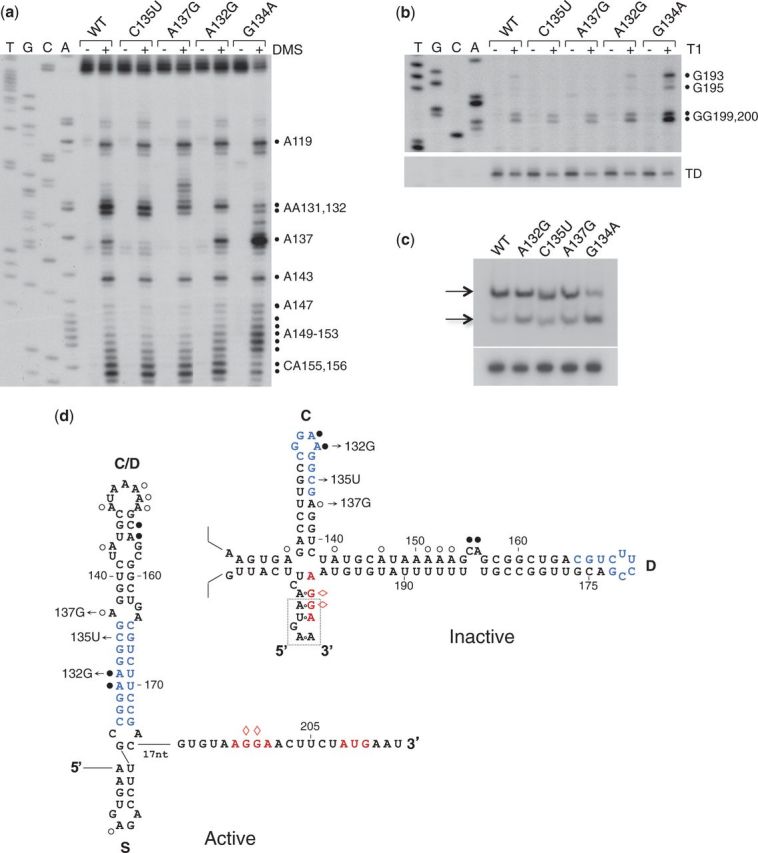
Structure determination of RNAs synthesized *in vitro* by T7 RNAP. RNA synthesized from P_T7_*-*PRE*-alx'* wild-type and mutants was treated with DMS (**a**) or RNase T1 (**b**). Reverse transcription of untreated (−) and DMS or T1 treated (+) RNA samples. The right panel (b; cleavage by T1) shows the region of the ribosome-binding site. In the lower part of this panel, a shorter exposure of a reverse transcription stop typical to the terminator at stem D (TD) is shown as a loading control. The numbers to the right indicate sequence positions relative to the transcription start site. (**c**) RNA as above was separated on non-denaturing polyacrylamide gels and transferred to nylon membranes by electroblotting (upper panel). To avoid detection of the short terminated transcripts, the membranes were probed with end-labeled primer complementary to sequences of *alx* (512). Arrows indicate the two conformations observed. To verify the integrity of this RNA, the samples were analyzed on denaturing 6% polyacrylamide, 7.8 M urea gel (lower part of the panel). (**d**) The relevant parts of PRE inactive and active conformations are shown with DMS modification data obtained with wild-type RNA. Circles indicate strong (filled circles) and weak (open circles) DMS modification sites. Red diamonds indicate T1 cleavage sites. The modification and the cleavage patterns of the pause site mutants resemble the patterns displayed by wild-type RNA. G_134_A mutant that forms the translationally active structure was used as a control.

The mutations could influence *alx* translation by affecting the dynamics of RNA folding *via* pausing or by changing the stability of one of the structures, shifting the equilibrium towards the alternative one. To rule out an effect on structure stability that could lead to changes in the ratio between the two forms, we probed the structure of RNAs synthesized *in vitro* using T7 RNAP. As opposed to *E. coli* RNAP, T7 RNAP exhibits much less pausing during elongation. In accordance with this feature, we showed previously that T7 RNAP could not promote formation of the active structure of PRE. The structures were probed under conditions of neutral pH, using DMS that methylates unpaired adenosine and cytidine residues at N1 and N3 positions, respectively, and by subjecting the RNAs to partial cleavage by RNase T1 that is specific for single-stranded guanosine residues. The modified nucleotides and the cleavage sites were mapped by primer extension (Figure 3a, b and d). As a control, we probed the structure of mutant G_134_A that was previously shown to maintain the active structure. In G_134_A RNA, residues A149–A153, located in loop C/D and A137 of the interior loop of this stem are much more accessible to DMS modification than in wild-type RNA. Conversely, in wild-type RNA that is predominantly found in the inactive form (14), the modification rates of residues C155, A156, located in the bulge of stem D and A131, A132 located in loop C of the inactive structure, are higher than in G_134_A mutant, where these nucleotides base pair forming hairpin C/D. Thus, residues A131, A132 and C155, A156 are indicative of the inactive form, whereas residues A149–A153 and A137 are indicative of the active form. Comparing the intensities of these modifications in wild-type and G_134_A RNAs with those of the mutants indicates that the modification pattern of the high-level expression pause site mutants resembles the pattern displayed by wild-type RNA, and the ratio of active to inactive forms is similar to that of the wild-type. Notably, in A_137_G RNA, the position A137 is no longer modified because of the mutation. Also, in C_135_U RNA, the position A137 can no longer be modified, possibly because of stabilization of stacking due to formation of pairs of UG/GU in tandem (21). In A_132_G RNA, the position A132 is no longer modified because of the A to G substitution, and A131 seems to be less accessible to DMS. However, the modification pattern of residues C155, A156 and A149–153 in these mutants resembles the pattern displayed by wild-type RNA, contrasting with the pattern displayed by the high-level expression mutant G_134_A. Furthermore, probing of the ribosome binding site by RNase T1 shows that the accessibility of this region in the pause site mutants is similar to that displayed by the wild-type, in contrast to the high-level expression mutant G_134_A, which forms almost exclusively the active structure (Figure 3b and d). In addition, to visualize the different conformers, we analyzed the mutant RNAs on non-denaturing polyacrylamide gels. Previously, we observed that the high-level expression mutant G_134_A RNA is found almost exclusively in one structure, whereas the majority of wild-type is found in the other conformation (14). The native gel shows that the distribution pattern of the mutant RNAs is similar to wild-type (Figure 3c). Taken together, the data show that when synthesized with limited pausing, the ratio between the active and inactive structures in the mutant RNAs is similar to that observed in the wild-type. These results indicate that the mutations influence translation of *alx* not because they affect structure stability, shifting the equilibrium in favor of one of the structures, but possibly because they modify pausing and thereby the dynamics of folding.

### The mutations modify the extent and/or position of pausing

**Figure 4.**
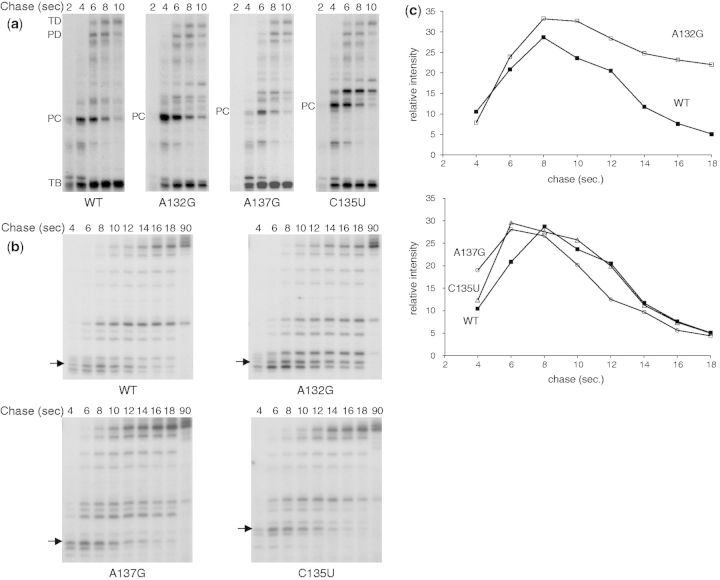
The mutations modulate pausing position and longevity. (**a**) Synchronized single round *in vitro* transcription of full-length PRE wild-type and mutant RNAs carried out using *E. coli* RNAP. After formation of initiation complexes, transcription was allowed to proceed for the indicated times (in seconds) by adding all four unlabeled NTPs in excess and rifampicin. TB and TD indicate RNA products generated by the *rho*-independent transcription terminators at hairpins B and D, respectively. PC and PD indicate pause sites at hairpins C and D, respectively. (**b**) Single round transcription reactions of truncated (Δ12–91) PRE using PCR generated templates were performed at 25°C as described in ‘Materials and Methods’ section. The last lane in each run indicates runoff transcripts obtained with PCR generated templates (10 sec. (a) and 90 sec (b)). Arrows indicate the position of pause C as verified by comparison with reactions using plasmid templates (Supplementary Figure S2). (**c**) Pause intensities of wild-type and mutants relative to total transcription are plotted as a function of time (chase).

To characterize the effect of the mutations on pausing, we monitored pausing in these mutants by synchronized single-round *in vitro* transcription (Figure 4a). These assays suggest that the position of pause C shifts forward in mutants A_137_G and C_135_U. For better resolution, we further characterized the effect of the mutations, using templates deleted for the majority of the upstream sequence including hairpins A and B (Δ12–91). As single point mutations can perturb separation that is based on RNA length, we estimated the position of pausing by using suitable RNA size markers, each carrying the corresponding point mutation. This mapping shows that mutations A_137_G and C_135_U shift the location of pause C two to three nucleotides forward, relative to wild-type, whereas in mutant A_132_G, the natural position of pause C is maintained (Supplementary Figure S1). The data showing that shifting the site forward is accompanied by a rise in *alx* translation endorse the mechanistic perception we previously suggested for *alx* regulation. Given that typically 12 ± 1 nucleotides of the nascent RNA are sequestered within the transcription elongation complex (22), RNAP, when paused at position G138, sequesters the sequence required to form the stem of hairpin C, from position G138 to C127 (Figure 2a). Once the formation of stem C is excluded, the free transcript preceding position C127 is capable of forming hairpin S (A114–U125). Shifting the site forward from position 138 and farther increases the chances of hairpin S to form and thereby stimulates the formation of the translatable structure.

Given that the high-level expression mutant A_132_G retains the natural position of pause C, we examined the effect of this mutation on the extent of pausing using linear templates. Single round transcription using linear templates enabled estimation of pause intensity relative to the total transcription as represented by the runoff. Mutant A_132_G exhibits increased pause intensity as well as extended pause duration (Figure 4b and c). As for pause duration, the decrease in the intensity of pausing in mutant A_132_G over a 10 s interval is significantly different from that of wild-type (33 and 83%, respectively). These results suggest that an increase in the rate of pausing by RNAP accompanied by a substantially slower pause escape increase the proportion of transcripts in which the formation of stem C is inhibited, thereby allowing the formation of the translationally active structure. Pause extent measurements of mutants A_137_G and C_135_U show that these mutants exhibit pause characteristics similar to wild-type (Figure 4b and c), suggesting that in these mutants the location of pausing plays a major role.

### Changes in transcriptional pausing influence RNA folding

**Figure 5.**
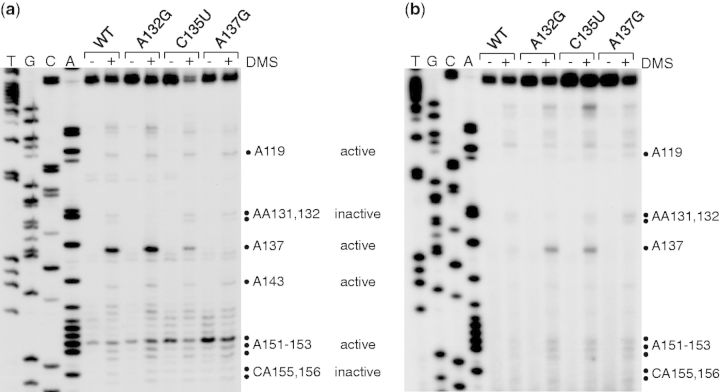
*In vitro* cotranscriptional and *in vivo* structure probing*.* (**a**) RNA was transcribed *in vitro* by *E. coli* RNAP for 5 min before the addition of DMS. (**b**) Cells containing plasmids carrying the locus P*_alx_*-PRE-*alx'* were grown in LB and treated with DMS for 5 min before RNA extraction. Reverse transcription of DMS modified (+) and unmodified (−) RNA. Modifications indicative of the active structure are marked as ‘active’, whereas modifications indicative of the inactive structure are marked as ‘inactive’. A summary of cotranscriptional structure probing data is displayed in Figure 2.

The differences in RNA folding caused by the changes in transcriptional pausing were investigated in assays of cotranscriptional structure probing. In these assays, the structures of the different mutant RNAs were determined using DMS during synthesis with *E. coli* RNAP (Figure 5a; summarized in Figure 2). To learn about changes in the proportion between the structures, active and inactive, we calculated the ratio of intensities of DMS modification of nucleotides that are indicative of the active or the inactive structures. Because in C_135_U and A_137_G mutants, the position A137 is no longer modified and in A_132_G RNA, position A131 seems to be less accessible to DMS, we measured the modification ratio of residues A153/C155 (indicative of the active and inactive structure, respectively). The calculated ratios in A_132_G, C_135_U and A_137_G RNAs are 2.8, 3.4 and 4.1, respectively, whereas wild-type RNA exhibits a ratio of 1.8. On the basis of these measurements, the active to inactive ratios of these high-level expression mutants are higher than that of wild-type RNA. Our results show that shifting the pause site farther from hairpin S or increasing the extent of pausing leads to an increase in the formation of the alternative, translationally active structure, correlating with the rise in expression observed *in vivo.*

### *In vivo* structure probing

To correlate the effect of the mutations on RNA folding as detected *in vitro* with RNA folding occurring *in vivo*, we compared the *in vivo* RNA structure of the mutants with wild-type. Notably, position A137 in mutant C_135_U is modified *in vivo* in contrast to the *in vitro* assays. The *in vivo* probing shows that the modification ratios of A137/A131 and A153/C155 increase (Figure 5b), thus demonstrating that the ratio of active to inactive structures in the high-level expression mutants shifts towards the translationally active structure. Taken together, the results demonstrate that transcription that involves pausing governs the process of PRE RNA folding, providing the mechanistic basis for PRE-*alx* regulation.

## DISCUSSION

In recent years, a large number of riboregulators have been discovered and characterized, of which, a subset that includes riboswitches affects expression in *cis*, by adopting different conformations in response to cellular and/or environmental signals (23,24). With respect to mechanisms, riboswitches can be assigned to two categories: thermodynamically driven and kinetically driven switches. The action of riboswitches with a thermodynamic regime is mainly governed by a thermodynamic equilibrium between ligand bound and unbound conformations. The conformation of such riboswitches changes post-transcriptionally on ligand binding or ligand dissociation. For example, in the *add* riboswitch of *Vibrio vulnificus*, the ribosome binding site of the downstream gene is sequestered in the absence of adenine. Following adenine binding, a structural rearrangement occurs that exposes the site to ribosomes, thereby leading to *add* translation. The conformational change conferred by adenine binding, as well as the rise in translation initiation, have been shown to occur *in vitro* with full-length *add* riboswitch transcripts, indicating that this adenine riboswitch is a thermodynamically driven switch (12).

In riboswitches with a kinetic regime, RNA folding and ligand binding occur cotranscriptionally. Cotranscriptional folding allows formation of structures that are often different from those displayed when a full transcript folds as a whole. These structures are in many cases not optimally stable. The process of cotranscriptional folding is influenced by two main determinants, transcription progression and transcription pausing. As transcription is directional and basepairing is fast compared with transcription rate, upstream sequences can basepair before downstream sequences have been transcribed or emerged from the polymerase exit channel (25,26). Transcriptional pausing is a temporary inhibition of transcript elongation. By halting the transcribing enzyme at strategic positions, it can affect regulatory interactions. In some cases, transcriptional pausing may prevent formation of competing structures, thus allowing the formation of complex, or less stable, structures (10,27). In other cases, pausing enables binding of ligand by extending the time frame available for interactions (11). One such example is the *ribD* riboswitch of *Bacillus subtilis*. In this kinetically driven riboswitch, an antiterminator stem that prevents premature termination forms in the absence of flavin mononucleotide (FMN). Conversely, cotranscriptional binding of FMN to *ribD* RNA results in formation of a transcription terminator. Two pause sites located between the aptamer and the expression platform delay the regulatory decision, extending the time frame for FMN binding (11). Another example of a kinetically driven switch is *pbuE* of *B. subtilis*, which depends on cotranscriptional binding of adenine to function (12,28). Interestingly, *pbuE* of *B. subtilis* and *add* of *V. vulnificus* both respond to adenine, yet their mechanistic properties differ. Whereas *pbuE* is kinetically driven*, add* is a thermodynamically driven genetic switch. Unlike the aforementioned examples of kinetically driven riboswitches that regulate transcription termination, the *E. coli btuB* riboswitch controls translation initiation in response to coenzyme B12 binding. Here, adequately positioned pause sites serve to isolate the aptamer region from the rest of the molecule so as to allow it to form a complex tertiary structure before downstream competing sequences become available (10).

The pH-responsive RNA element of *alx* is a riboregulator displaying kinetic properties. Its action depends on transcriptional pausing and cotranscriptional folding. The location of the pause sites led us to suggest that the paused enzyme sequesters sequences required for the formation of the stable, translationally inactive structure, thus allowing formation of a less stable, however translationally active structure. Here, we show that changing the position of the first pause site influences the regulatory result dramatically. Shifting the pause site farther from stem S increases the likelihood of hairpin S assembly, a requisite for the formation of the active structure. Consequently, the probability of the active structure to form is increased. Mutations A_137_G and C_135_U shift the position of PC from 138 to 140 and 141, respectively. These mutations increase *alx* expression by 2–6-fold. Because no other changes in pause characteristics can be detected in these mutants, the increase in *alx* expression is most probably due to the new location of pausing.

Mutant A_132_G pauses at the same position as wild-type. However, it exhibits a dramatic effect on the rate of decay; pause intensity in this mutant decreases slowly compared with wild-type. A_132_G mutant also shows a higher proportion of pausing. Both characteristics can explain the rise in *alx* translation. An increase in the number of transcription complexes paused at PC, as well as an extension in the time these complexes spend at that site, can both lead to an increase in the formation of stem S, and thus the active structure.

Mutations like A_132_G that extends the duration of pausing at PC and A_137_G and C_135_U that shift the position of PC, increase the probability of formation of stem S and thus the translationally active structure. The physiological and molecular characteristics of these mutations substantiate the model that describes the role of pH in this system, whereby high pH extends pausing at PC, thus leading to activation of *alx* translation. It is interesting to note that the response of these mutants to high pH is more moderate than that of wild-type, suggesting that mutations affecting pause behavior are less susceptible to the pH effects on pausing, either because of their direct effect on pause characteristics or due to an effect on the pH sensing mechanism.

Taken together, the mutational data illustrate the mechanistic design of *alx* regulation that relies on precise temporal and spatial characteristics, demonstrating the importance of both the precise location and the extent of pausing to RNA folding and control of gene expression. We propose that the mechanistic design of the *alx* regulatory RNA element is based on a delicate balance that provides an opportunity for environmental signals such as pH to introduce structural changes in the RNA and thereby modulate expression.

## SUPPLEMENTARY DATA

Supplementary Data are available at NAR Online, including [29].

## FUNDING

This work was supported by the Israel Science Foundation founded by The Israel Academy of Sciences and Humanities [911/09]; by Deutsche Forschungsgemeinschaft research grant [VO 875/5-1]; and by Israel Centers of Research Excellence (ICORE), Chromatin and RNA [1796/12]. Funding for open access charge: The Israel Science Foundation founded by The Israel Academy of Sciences and Humanities [911/09] and Deutsche Forschungsgemeinschaft research grant [VO 875/5-1].

*Conflict of interest statement*. None declared.

## Supplementary Material

Supplementary Data
