# Supplementary material for: Changes in transcriptional pausing modify the folding dynamics of the pH-responsive RNA element
Source: Nucleic Acids Res. 2013 Sep 26;42(1):622–30. doi: 10.1093/nar/gkt868 (PMC3874183; doi:10.1093/nar/gkt868)
Supplement: Supplementary Data [file supp_42_1_622__index.html]

Changes in transcriptional pausing modify the folding dynamics of the pH-responsive RNA element — Changes in transcriptional pausing modify the folding dynamics of the pH-responsive RNA element — Supplementary Data 

# Changes in transcriptional pausing modify the folding dynamics of the pH-responsive RNA element

## Supplementary Data

files

**Files in this Data Supplement:**

- Supplementary Data - pdf file
