# Supplementary material for: Changes in transcriptional pausing modify the folding dynamics of the pH-responsive RNA element
Source: Nucleic Acids Res. 2013 Sep 26;42(1):622–30. doi: 10.1093/nar/gkt868 (PMC3874183; doi:10.1093/nar/gkt868)
Supplement: Supplementary Data [file supp_gkt868_nar-01720-r-2013-File011.pdf]

SUPPLEMENTARY DATA

Changes in transcriptional pausing modify the folding dynamics of the pH-responsive RNA element

Gal Nechooshtan, Maya Elgrably-Weiss and Shoshy Altuvia*

Department of Microbiology and Molecular Genetics, IMRIC,

The Hebrew University-Hadassah Medical School, Jerusalem 91120, Israel.

***Correspondence**: shoshy.altuvia@mail.huji.ac.il

SUPPLEMENTARY RESULTS

(a)

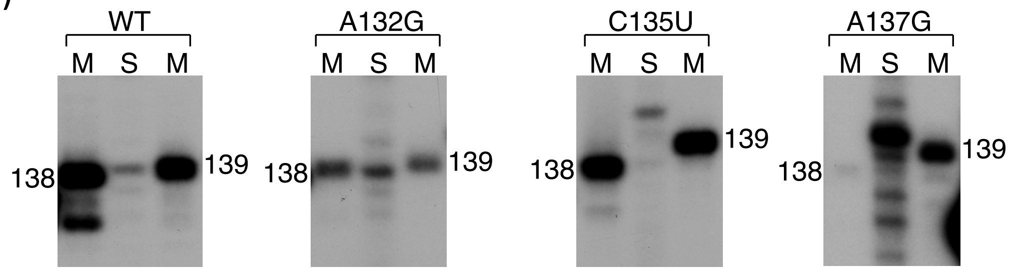

(b)

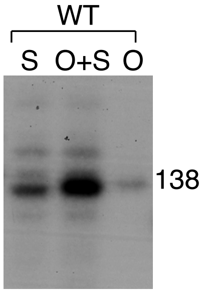

Figure S1. Localization of stem C pause site. (**a**) Single round transcription using wild type and mutant PRE templates deleted for bases 12-91. RNA markers, each carrying the corresponding mutation, were synthesized as described in Supplementary materials and methods. Single round transcription products (S). RNA size markers (M). (**b**) Localization of wild type stem C pause site by comparison with a custom made RNA oligonucleotide. Single round transcription reaction (S) was performed using pSA64 deleted for bases 12-109 of PRE as template. Custom made RNA oligonucleotide (O) carrying PRE nucleotides 2-11 followed by 110-138 was end labeled as described below. (O +S) Samples of both oligonucleotide RNA marker and single round transcription product were loaded in the same lane for accurate determination of pause position.

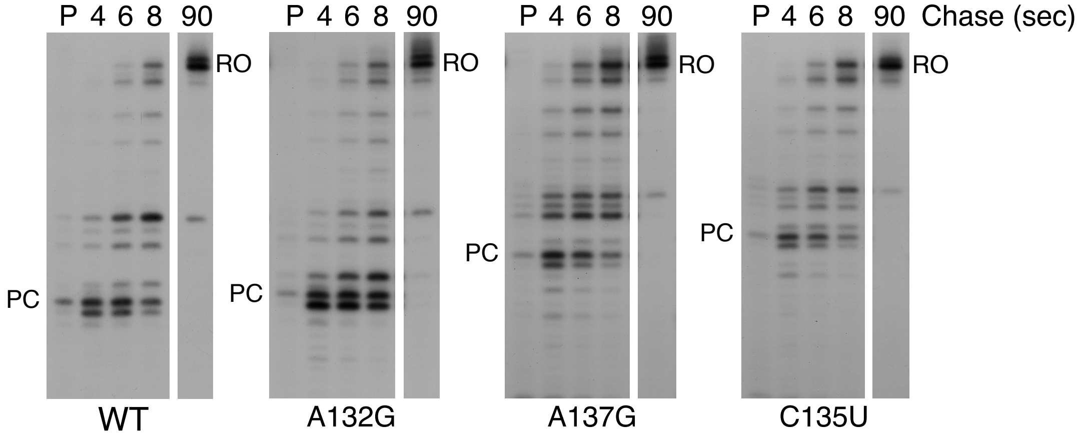

Figure S2. Single round transcription reactions of truncated (Δ12-91) PRE using plasmid (P) or PCR generated templates (4, 6, 8 and 90 sec) were performed as described in Materials and methods. Runoff transcripts obtained with PCR generated templates (RO).

SUPPLEMENTARY MATERIALS AND METHODS

Pause site localization

The stem C pause sites in wild type and mutants were localized by comparing single round transcription reactions to RNA markers, each carrying the corresponding mutation. Single round transcription reactions were performed essentially as described in Materials and methods, except that pSA64 deleted for bases 12-91 of PRE (wild type and mutants) was used as template, reactions were conducted at 30°C, the incubation time for formation of halted elongation complexes was 15 minutes, and plasmid and enzyme concentrations were doubled. The reactions were stopped after 4 seconds of chase. RNA markers were synthesized using PCR-generated templates carrying the T7 promoter directing the synthesis of nucleotides 2-11 of PRE followed by nucleotides 92-138 or 92-139 of PRE (T7 transcription was designed to start at +2, to correspond with the start site in our single round transcription assays). To reduce nontemplated nucleotide addition at the 3' termini of RNAs by T7 RNA polymerase, templates used to synthesize the RNA markers carried 2'-O-methyl modifications at the last two nucleotides of the minus strand 5' termini (29). Synthesis was performed in 20 μl reactions containing 40 mM Tris-HCl (pH 7.9), 6 mM $MgCl_2$, 10 mM dithiothreitol (DTT), 2 mM spermidine, 8 units RNase inhibitor (Takara Bio), 500 μM each CTP, GTP and UTP, 100 μM ATP, 6 μCi of α-^{32}P-ATP, 35-195 ng of gel-purified PCR product as template, and 10 U T7 RNA polymerase (New England Biolabs). The reactions were incubated for 2 hours at 37°C, followed by phenol/chloroform extraction. Thereafter, the reactions were purified on G50 columns (GE healthcare) as per the manufacturer's protocol, concentrated by SpeedVac and then dephosphorylated in 20 μl reactions containing 10 mM Tris-HCl (pH 7.5), 10 mM $MgCl_2$, 0.1 mg/ml BSA and 1 U shrimp alkaline phosphatase (Fermentas). After 30 minutes at 37°C the reactions were deactivated for 15 minutes at 65°C and drop dialyzed against double distilled water on 0.025μ VSWP filters (Millipore) for 30 minutes. Samples were analyzed on 8% polyacrylamide, 7.8 M urea gels.

In addition we reconfirmed the localization of wild type PC by comparison with a custom made RNA oligonucleotide. A single round transcription reaction was performed as described in Materials and methods, except that 2 μg of pSA64 deleted for bases 12-109 of PRE and 0.8 U of enzyme were used. After 2 seconds of chase the reaction was frozen in liquid nitrogen, phenol/chloroform extracted, and purified on a G50 column as per the manufacturer's protocol. Thereafter, half of the reaction was phosphorylated in a 50 μl reaction including 1mM ATP, 70 mM Tris-HCl (pH 7.6), 10 mM $MgCl_2$, 5 mM DTT and 1 μl (10 U) T4 polynucleotide kinase (PNK; New England Biolabs). The reaction was incubated for 30 minutes at 37°C. 90 pmol of an RNA oligonucleotide including nucleotides 2-11 of PRE followed by nucleotides 110-138 of PRE (1901; 5'-rGrCrA rArArG rGrGrG rArUrU rGrUrA rArGrU rGrArG rArCrC rUrUrG rCrCrG rGrArA rGrGrC rGrArG-3') were phosphorylated in a 15 μl reaction including 20 μCi of γ -^{32}P-ATP, 70 mM Tris-HCl (pH 7.6), 10 mM $MgCl_2$, 5 mM DTT and 5 U PNK. The reaction was incubated for 30 minutes at 37°C. Samples were analyzed on 8% polyacrylamide, 7.8 M urea gels.

Table S1. Plasmids

Plasmid	Construction[a]	Genetic elements[b]
pSA60	pRS552 (693-694)	P_{alx}-PRE-*alx*'-'*lacZ*
pSA60 $A_{132}G$	pRS552 (693-694)	P_{alx}-PRE_{A132G}-*alx*'-'*lacZ*
pSA60 $A_{137}G$	pRS552 (693-694)	P_{alx}-PRE_{A137G}-*alx*'-'*lacZ*
pSA60 $C_{135}U$	pRS552 (693-694)	P_{alx}-PRE_{C135U}-*alx*'-'*lacZ*
pSA64	pGEM4 (1004-1005)	P_{alx}-PRE-*alx*'
pSA64 $A_{132}G$	pGEM4 (1004-1005)	P_{alx}-PRE_{A132G}-*alx*'
pSA64 $A_{137}G$	pGEM4 (1004-1005)	P_{alx}-PRE_{A137G}-*alx*'
pSA64 $C_{135}U$	pGEM4 (1004-1005)	P_{alx}-PRE_{C135U}-*alx*'
pSA64 Δ12-91	pGEM4 (1004-1005)	P_{alx}-PRE Δ12-91-*alx*'
pSA64 Δ12-91 $A_{132}G$	pGEM4 (1004-1005)	P_{alx}-PRE Δ$12\text{-}91_{A132G}$-*alx*'
pSA64 Δ12-91 $A_{137}G$	pGEM4 (1004-1005)	P_{alx}-PRE Δ$12\text{-}91_{A137G}$-*alx*'
pSA64 Δ12-91 $C_{135}U$	pGEM4 (1004-1005)	P_{alx}-PRE Δ$12\text{-}91_{C135U}$-*alx*'
pSA64 Δ12-109	pGEM4 (1004-1005)	P_{alx}-PRE Δ12-109-*alx*'
pSA65	pZE12 (1361-1362)	P_{alx}-PRE-*alx*'
pSA65 $A_{132}G$	pZE12 (1361-1362)	P_{alx}- PRE_{A132G}-*alx*'
pSA65 $A_{137}G$	pZE12 (1361-1362)	P_{alx}- PRE_{A137G}-*alx*'
pSA65 $C_{135}U$	pZE12 (1361-1362)	P_{alx}- PRE_{C135U}-*alx*'

pSA66	pGEM4 (940-1005)	P_{T7}-PRE-*alx*'
pSA66 $G_{134}A$	pGEM4 (940-1005)	P_{T7}-PRE_{G134A}-*alx*'
pSA66 $A_{132}G$	pGEM4 (940-1005)	P_{T7}-PRE_{A132G}-*alx*'
pSA66 $A_{137}G$	pGEM4 (940-1005)	P_{T7}-PRE_{A137G}-*alx*'
pSA66 $C_{135}U$	pGEM4 (940-1005)	P_{T7}-PRE_{C135U}-*alx*'

[a]The numbers in parentheses represent primers used for fragment amplification
[b]Fragments end at nucleotide 260 of PRE-*alx* RNA.

Table S2. Oligonucleotides

Oligonucleotides used for plasmid construction

Primer	Primer sequence (5'-3')	Fragment[a]
693	GCGAATTCTGGATGAGCATCTGGTG	P_{alx}-PRE-*alx*' (+)
694	GTGGATCCGCGACAACAACAGCGA	P_{alx}-PRE-*alx*' (-)
940	CGCAAGCTTTGCAAAGGGGAGTAACTTCATTGCC	PRE-*alx*' (+)
1004	CGCAAGCTTTGGATGAGCATCTGGTGG	P_{alx}-PRE-*alx*' (+)
1005	GCGAATTCGCGACAACAACAGCGAATC	P_{alx}-PRE-*alx*' (-)*
1361	CGCTCGAGTGGATGAGCATCTGGTG	P_{alx}-PRE-*alx*' (+)
1362	GCTCTAGAGCGACAACAACAGCGA	P_{alx}-PRE-*alx*' (-)

[a]Plus (+) and minus (-) strands are indicated.
*Primer 1005 was also used for generation of the PRE-*alx*' fragment.

Oligonucleotides used for synthesis of PCR-generated templates for transcription

Primer	Primer sequence (5'-3')[a]	Fragment[b]	Fragment end location
1836	mCmUCGCCTTCCGGCAAGG	P_{T7}-PRE' Δ12-91 (-)	Nucleotide 138 of PRE
1856	mCmCTCGCCTTCCGGCAAG	P_{T7}-PRE' Δ12-91 (-)	Nucleotide 139 of PRE
1906	TCAGCCGCTGCTTTTTATG	P_{alx}-PRE' Δ12-91 (-)*	Nucleotide 164 of PRE
1913	CGAAATTAATACGACTCACTATAGCAAAGGGGAAGACGTATTC	P_{T7}-PRE' Δ12-91 (+)	
1914	mCmUCGCCCTCCGGCAAG	P_{T7}-PRE' Δ12-91_{A132G} (-)	Nucleotide 138 of PRE
1915	mCmCTCGCCCTCCGGCAAG	P_{T7}-PRE' Δ12-91_{A132G} (-)	Nucleotide 139 of PRE
1916	mCmUCACCTTCCGGCAAG	P_{T7}-PRE' Δ12-91_{C135U} (-)	Nucleotide 138 of PRE
1917	mCmCTCACCTTCCGGCAAG	P_{T7}-PRE' Δ12-91_{C135U} (-)	Nucleotide 139 of PRE
1919	mCmCCGCCTTCCGGCAAG	P_{T7}-PRE' Δ12-91_{A137G} (-)	Nucleotide 138 of PRE
1920	mCmCCCGCCTTCCGGCAAG	P_{T7}-PRE' Δ12-91_{A137G} (-)	Nucleotide 139 of PRE

[a]nucleotides preceded by m are 2'-O-methyl modified.

[b]2'-O-methyl modified primers were used with primer 1913 to generate templates for synthesis of RNA size markers. Plus (+) and minus (-) strands are indicated.

*Primers 1004 and 1906 were used to generate linear templates for Pause C kinetic analysis.

Oligonucleotides used for site-directed mutagenesis		
Primer	Primer sequence (5'-3')	Mutation[a]
1118	GCATAGACCCCGCCTTCCGGCAAGGTC	$A_{137}G$ (-)
1119	GAAGGCGGGGTCTATGCATAAAAAGCAGCGG	$A_{137}G$ (+)
1619	CCGGCAAGGTCTCACTTAC	$A_{132}G$; $C_{135}U$ (-)
1621	AGGGCGAGGTCTATGCATAAAAAGC	$A_{132}G$ (+)
1622	AAGGTGAGGTCTATGCATAAAAAGCAGCG	$C_{135}U$ (+)
1849	TCCCCTTTGCAGGTAAC	Δ12-109; Δ12-91 (-)
1850	TTGTAAGTGAGACCTTGCC	Δ12-109 (+)
1905	AGACGTATTCCTTTTTTGTTG	Δ12-91 (+)

[a]Plus (+) and minus (-) strands are indicated.

Oligonucleotides used for primer extension*		
Primer	Primer sequence (5'-3')	5' end location
512	GGCGACAACAACAGCGA	Nucleotide 261 of PRE-*alx*
1048	CATAGAAGTTCCTTACACATAAA	Nucleotide 210 of PRE-*alx*

*All primer extension reactions were performed with primer 1048
except for RNase T1 analysis, where 512 was used. Primer 512 was also used for native gel analysis.

SUPPLEMENTARY REFERENCES

29. Kao,C., Zheng,M. and Rudisser,S. (1999) A simple and efficient method to reduce nontemplated nucleotide addition at the 3 terminus of RNAs transcribed by T7 RNA polymerase. *RNA*, **5**, 1268-1272.
